# Anesthetic Management of Brain-Dead Donors During Organ Retrieval: Hemodynamic Effects and Potential Organ-Protective Implications – A Retrospective Analysis of 85 Cases

**DOI:** 10.3389/ti.2026.16262

**Published:** 2026-04-21

**Authors:** Jan Sönke Englbrecht, Tobias Piegeler, Mira Küllmar, Christopher Marschall, Svitlana Ziganshyna

**Affiliations:** 1 Department of Anesthesiology, Intensive Care Medicine and Pain Therapy, University Hospital Münster, Münster, Germany; 2 Department of Anesthesiology and Intensive Care, University of Leipzig Medical Center, Leipzig, Germany; 3 Organ Donation Coordinator Unit, University of Leipzig Medical Center, Leipzig, Germany

**Keywords:** anesthesia, brain death, donor management, hemodynamics, organ retrieval

## Abstract

Currently, no evidence-based recommendations for anesthetic management of brain-dead organ donors exist. Hemodynamic responses to surgical stimulation and potential organ-protective effects of anesthetic agents have been reported inconsistently. We retrospectively analyzed anesthetic management of all donors at University Hospital Münster between 2010 and 2025. Heart rate (HR) and mean arterial pressure (MAP) were assessed before, during, and up to 15 min after first incision. Eighty-five donors were included; volatile anesthetics were administered in 41%, opioids in 80%, and neuromuscular blocking agents in 92%. HR (bpm) remained unchanged from before (94 [85–105]) to during (93 [84–104]) and post-incision (95 [85–103]). MAP (mmHg) decreased from 5 minutes (86 [76–95]) to 15 min post-incision (80 [72–89]; p = 0.034). Sufentanil did not affect HR or MAP at any point. Sevoflurane was associated with lower HR at all time points (p < 0.001) and lower MAP during incision (p = 0.020), but independent of surgical stimulation. Anesthetic management varied substantially. Hemodynamics did not increase following incision, and our findings do not support opioid administration, whereas hemodynamic effects of sevoflurane must be carefully managed to ensure sufficient organ perfusion during retrieval. Evidence-based recommendations for anesthetic management are needed to support organ-protective strategies in organ donation.

## Graphical Abstract

**Figure:**
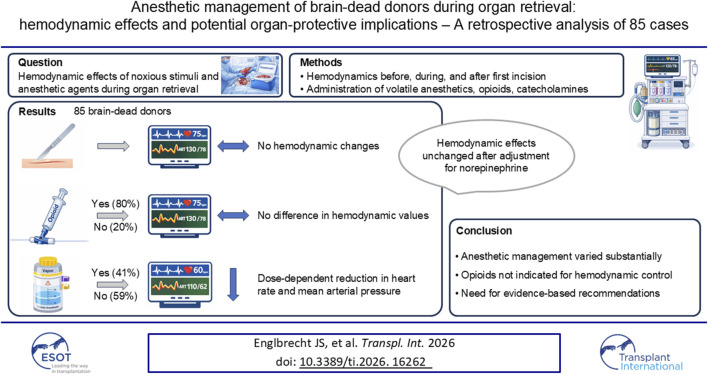

## Introduction

The supply of transplantable organs remains insufficient worldwide [1]. Thus, adequate donor management is essential to increase the number and quality of available organs for donation [2]. However, evidence-based recommendations for organ-protective measures during intensive care unit (ICU) stay and organ retrieval for donation after brain death (DBD) donors are very limited. Due to the paucity of available studies, recommendations for ICU therapy are mainly based on pathophysiological reasoning, epidemiological observations, or extrapolations from general ICU management strategies [2, 3]. Surprisingly few studies have examined the role and influence of anesthesia during organ retrieval [4, 5]. Accordingly, no evidence-based recommendations are currently available regarding the anesthetic management of DBD donors [4–6]. This results in non-standardized management, especially concerning the indications for the administration of anesthetic agents, as well as targeted hemodynamic values and indication for and selection of catecholamines [4, 7, 8].

Previous studies have demonstrated that DBD donors exhibit both a hemodynamic reaction with increase in heart rate (HR) and mean arterial pressure (MAP), and an endocrine stress reaction to surgical stimuli [9–12]. It has been suggested that volatile anesthetics might attenuate these responses [13]. However, a recent study reported no consistent changes in HR or MAP in donors receiving anesthetic agents at the time of skin incision [4]. In addition to their possible hemodynamic effects, volatile anesthetics have been suggested to mitigate ischemia–reperfusion injury (IRI), a critical pathological process and an unavoidable challenge in organ transplantation. Thus, the use of volatile anesthetics during organ retrieval may offer potential organ-protective benefits through anesthetic preconditioning beyond possible hemodynamic effects [6, 14–17].

The indication for opioids in this context is even less well studied. The limited available evidence suggests that opioids do not significantly alter hemodynamic responses or the release of stress hormones due to surgical stimuli under these conditions [18, 19]. Some authors thus recommend avoiding their use during organ retrieval [6], while others support it [20]. Whether opioids influence IRI in the context of transplantation remains unknown [21]. The available evidence is derived mainly from animal studies demonstrating, for example, a reduction in acute renal injury mediated by pharmacological preconditioning with opioids [22].

Additionally, administering anesthetic agents to brain-dead patients is a matter of an ongoing ethical debate. While the German Ethics Council states that these drugs are unnecessary in DBD donors [23], some authors argue that using anesthetic agents during organ retrieval represents an ethical obligation to ensure the patient’s dignity and to alleviate the moral distress of clinicians faced with autonomic reflexes to nociceptive stimuli [24, 25].

The present study therefore aimed to address two key questions. First, whether the administration of volatile anesthetics and opioids in DBD donors might influence a possible hemodynamic response to surgical stimuli. Second, how anesthesiologists manage organ retrieval, particularly regarding the use of anesthetic agents and hemodynamics.

## Patients and Methods

This study was performed in accordance with the Declaration of Helsinki. The Ethics Committee of the University of Muenster approved the study protocol (file number 2021-801-f-S) on 28 May 2025. The need for informed consent was waived due to the retrospective analysis of routinely collected patient data.

We retrospectively identified all utilized DBD donors at University Hospital Münster from January 2010 to October 2025 and included all donors aged ≥14 years into further analysis. For each case, basic demographic parameters (sex, age, height, weight) and the etiology of the devastating brain injury were obtained through review of the medical records.

To analyze hemodynamics and anesthetic management, five time points during organ retrieval were defined:

- pre (baseline, 5 min before first incision)
- 0 min (during first incision)
- 5 min, 10 min, 15 min (five, 10, and 15 min after first incision)

The following parameters were extracted from the anesthesia records at each time point:

- HR (bpm)
- MAP (mmHg)
- Type and infusion rate of administered catecholamines
- Average end-tidal concentration of volatile anesthetics from pre to 15 min

Additionally, the type and cumulative dose of administered opioids and neuromuscular blocking agents (NMBA) from 30 min before until first incision (0 min) were recorded.

### Statistical Methods

All statistical analyses were performed using SPSS (IBM, Version 31). Additional verification of results and figure generation were conducted in Python (version 3.11). Normality of all continuous variables was assessed using the Shapiro–Wilk test. Data following a normal distribution were expressed as mean ± SD, and non-normally distributed data as median [IQR, 25th-75th percentile]. Within-subject comparisons of continuous variables across time points were conducted using repeated-measures ANOVA for normally distributed variables or Friedman test for non-normally distributed data. Post-hoc pairwise comparisons were performed using paired *T*-tests or Wilcoxon signed-rank tests, respectively. Between-group comparisons were analyzed using Mann–Whitney U tests for both absolute values and relative changes from baseline (baseline = pre, Δ values). To increase group sizes and reduce the number of necessary comparisons, the end-tidal concentrations of desflurane and isoflurane, which were used in only a few cases, were converted into equipotent sevoflurane concentrations [26]. Associations between continuous variables were assessed using Spearman rank correlation for both absolute and Δ values. To evaluate potential additive or interaction effects of volatile anesthetics and opioids on HR and MAP, a two-way analysis of variance was performed with both agents as factors and a nonparametric aligned-rank transform ANOVA was applied. Both absolute values and Δ values were analyzed at each time point. To account for potential confounding by catecholamine administration, time-matched partial Spearman correlations were computed, evaluating the relationship of catecholamine infusion rate and hemodynamic parameters while controlling for opioid dose and volatile anesthetics concentration, and *vice versa*. All variables were rank-transformed before partial correlation, and residuals after regression on covariates were correlated to yield partial Spearman’s ρ. All tests were two-tailed. To control for multiple testing, Bonferroni correction was applied, and a corrected p ≤ 0.05 was considered statistically significant. The detailed description and results of all subsequent statistical analyses are provided in the Supplementary Material.

## Results

In total, 96 DBD donors were utilized between January 2010 and October 2025 at our institution. Six cases were excluded due to age at admission (<14 years), and an additional five cases due to missing data. Accordingly, 85 cases were included in the further analysis (Table 1). The minimum monitoring standard for all donors during organ retrieval comprised ECG, pulse oximetry, invasive arterial blood pressure monitoring, and central venous catheterization.

**TABLE 1:** Demographics of the study cohort.

Parameter	value
Male/female, *n* (%)	56/29 (66/34)
Age (years)	45.0 [31.5–63.0]
Height (cm)	177 ± 8.7
Weight (kg)	80.0 [70.0–90.0]
Etiology of devastating brain injury Intracranial hemorrhage, *n* (%) Hypoxic brain injury, *n* (%) Traumatic brain injury, *n* (%) Stroke, *n* (%) Others, *n* (%)	31 (36) 28 (33) 20 (24) 4 (5) 2 (2)

Data are presented as mean ± SD, in normally distributed data, otherwise as median [IQR].

### Anesthetic Management During Organ Retrieval

Since no evidence-based recommendations are available, management was mainly determined by the attending senior anesthesiologist. Volatile anesthetics were administered in 41% DBD donors during organ retrieval, with sevoflurane used in all but three cases. No intravenous hypnotic agents were used in any of the cases. Sufentanil was the only opioid used and was administered in 80% of the cases prior to incision, while NMBA were used prior to incision in 92% (Table 2). At least one catecholamine was used in 88% of the cases (Table 3).

**TABLE 2:** Administered anesthetic agents during organ retrieval.

Anesthetic agent	“No”, *n* (%)	“Yes”, *n* (%)	Dosage
Volatile anesthetics - Sevoflurane - Desflurane - Isoflurane	50 (59)	35 (41) 32 (91) 2 (6) 1 (3)	1.2 ± 0.6* 3.9* 0.7*
Opioids - Sufentanil	17 (20)	68 (80) 68 (100)	0.56 μg kg^−1^ [0.31–0.67]
Neuromuscular blocking agents - Cisatracurium - Rocuronium	7 (8)	78 (92) 46 (59) 32 (41)	0.17 mg kg^−1^ [0.13–0.24] 0.86 mg kg^−1^ [0.65–1.25]

Data are presented as mean ± SD, in normally distributed data, otherwise as median [IQR]. *mean end-tidal concentration (%) from pre to 15 min after incision.

**TABLE 3:** Distribution of administered catecholamines during organ retrieval.

Agent	Administered, n (%)	Norepinephrine	Epinephrine	Dobutamine	Dopamine	Vasopressin
None, *n* (%)	10 (12)	-	-	-	-	-
One, *n* (%)	56 (66)	49	0	1	1	5
Two, *n* (%)	14 (16)	14	3	2	2	7
Three, *n* (%)	5 (6)	5	2	2	1	5
Total, *n* (%)	85 (100)	68	5	5	4	17

Type and number of administered catecholamines.

Infusion rates of catecholamines were analyzed across all time points. Group sizes for epinephrine, dobutamine, and dopamine were small, and therefore the Friedman test was used as a more robust approach although distributions appeared to be normal. Only vasopressin infusion rates showed a significant difference over time (p = 0.016, partial η^2^ = 0.171). However, *post hoc* analysis showed no significant differences between time points (p ≥ 0.206 for all, Cohen´s d_z_ = 0.243–0.512) (Table 4; Supplementary Table S1).

**TABLE 4:** Dosage of administered catecholamines during organ retrieval.

Substance	*n (%)*	pre	0 min	5 min	10 min	15 min
Norepinephrine (µg kg^-1^ min^-1^)	68 (80)	0.071 [0.021–0.154]	0.072 [0.024–0.154]	0.074 [0.021–0.143]	0.073 [0.026–0.143]	0.071 [0.029–0.143]
Epinephrine (µg kg^-1^ min^-1^)	5 (6)	0.083 ±0.042	0.081 ±0.045	0.081 ±0.045	0.085 ±0.045	0.084 ±0.042
Dobutamine (µg kg^-1^ min^-1^)	5 (6)	2.667 ±1.979	2.667 ±1.979	2.667 ±1.979	3.167 ±1.354	3.167 ±1.354
Dopamine (µg kg^-1^ min^-1^)	4 (5)	2.412 ±0.626	2.412 ±0.626	2.412 ±0.626	2.412 ±0.626	2.412 ±0.626
Vasopressin (I.E., h^-1^)	17 (20)	1.629 ±0.696	1.600 ±0.712	1.559 ±0.743	1.541 ±0.774	1.541 ±0.774

Data are presented as mean ± SD, in normally distributed data, otherwise as median [IQR]. Infusion rates over time from baseline (pre), during incision (0 min) until 15 min after incision.

### Hemodynamics During Organ Retrieval

**FIGURE 1:**
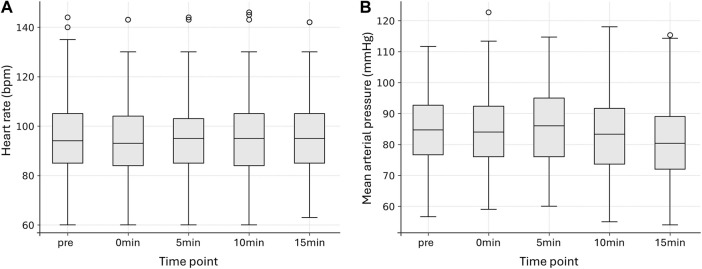
Hemodynamics during organ retrieval. Boxplots showing heart rate (bpm) **(A)** and mean arterial pressure (mmHg) **(B)** at baseline (pre), during incision (0min), and 5, 10, and 15 min after incision. Boxes represent the IQR, horizontal lines denote the median, and whiskers indicate values within 1.5 × IQR. Open circles represent data points classified as outliers (beyond 1.5 × IQR).

HR remained stable throughout the period from before incision (pre) until 15 min after incision (Figure 1A; Supplementary Table S2). There were no significant differences between time points (p = 0.308, Kendall´s W = 0.014). A significant difference in MAP was found across all time points (p = 0.007, Kendall´s W = 0.042), with *post hoc* analysis revealing only a modest decrease at 15 min compared with 5 min after incision (p = 0.034, r = −0.317) (Figure 1B; Supplementary Table S2). Six (7%) DBD donors exhibited a MAP <65 mmHg at one time point, seven (8%) at two consecutive time points, and two (2%) at four time points.

### Effects of Sufentanil on Heart Rate and Mean Arterial Pressure

**FIGURE 2:**
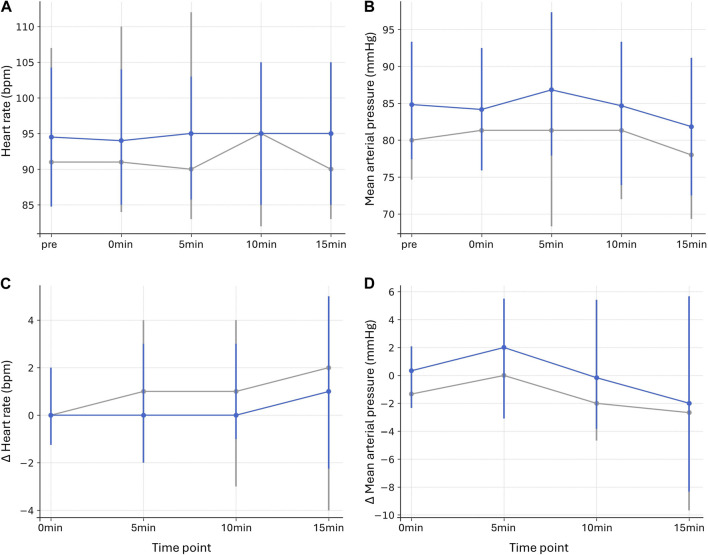
Heart rate and mean arterial pressure in donors with and without sufentanil Heart rate (bpm). **(A)** and mean arterial pressure (mmHg) **(B)** at baseline (pre), during incision (0min), and 5, 10, and 15 min after incision. Changes from baseline (pre, Δ values) are displayed for heart rate **(C)** and mean arterial pressure **(D)** for corresponding time points. Data are presented as median values with interquartile ranges (vertical lines). Gray lines represent cases without, and blue lines with sufentanil administration.

Sufentanil administration showed no significant influence on either HR or MAP at any time point. Across all comparisons, median absolute HR values were comparable between donors with and without sufentanil (p = 1.000 for all, r = −0.092 to 0.024) (Figure 2A; Supplementary Table S3). For absolute MAP values, no time point reached significance, though a weak tendency toward higher values at 15 min in the group with sufentanil was observed (p = 0.345, r = −0.287) (Figure 2B; Supplementary Table S3). Changes from baseline HR (Δ HR, p = 1.000 for all, r = −0.116 to 0.064) and MAP (Δ MAP, p ≥ 0.600 for all, r = −0.228 to −0.156) were likewise similar between groups (Figures 2C,D; Supplementary Table S3).

Correlation analyses revealed no clear dose-dependent effects of sufentanil on HR or MAP when considering absolute values (p ≥ 0.754 for all, ρ = −0.003–0.138). When changes relative to baseline were analyzed, no time point reached statistical significance for Δ HR (p ≥ 0.919 for all, ρ = −0.073–0.132). For Δ MAP, a weak positive association between sufentanil dose and Δ MAP was observed at 0 min (p = 0.079, ρ = 0.252) and 10 min (p = 0.094, ρ = 0.245) (Supplementary Table S3).

### Effects of Sevoflurane on Heart Rate and Mean Arterial Pressure

In contrast, sevoflurane administration was associated with consistently lower HR and MAP.

**FIGURE 3:**
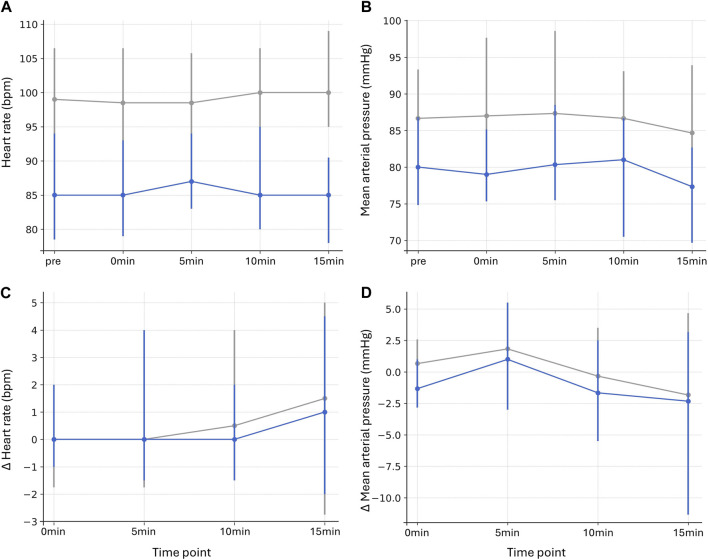
Heart rate and mean arterial pressure in donors with and without sevoflurane Heart rate (bpm). **(A)** and mean arterial pressure (mmHg) **(B)** at baseline (pre), during incision (0min), and 5, 10, and 15 min after incision. Changes from baseline (pre, Δ values) are displayed for heart rate **(C)** and mean arterial pressure **(D)** for corresponding time points. Data are presented as median values with interquartile ranges (vertical lines). Gray lines represent subjects without, and blue lines with sevoflurane.

Absolute HR values were significantly lower in the sevoflurane group at all time points (p ≤ 0.001 for all, r = 0.470–0.571). Absolute MAP values were likewise reduced, but only significantly at 0 min (p = 0.020, r = 0.386), and with trends at pre (p = 0.093, r = 0.302), 5 min (p = 0.114, r = 0.292), 10 min (p = 0.075, r = 0.312), and 15 min (p = 0.057, r = 0.325), respectively (Figures 3A,B; Supplementary Table S4). No significant changes were observed for Δ HR (p = 1.000 for all, r = −0.114 to 0.048) or Δ MAP (p ≥ 0.193 for all, r = 0.030–0.253) at any time point (Figures 3C,D; Supplementary Table S4).

Dose–response analyses confirmed a negative correlation between sevoflurane concentration and both HR and MAP. For absolute HR values, correlations were strongly negative across all time points (p < 0.001 for all, ρ = −0.499 to −0.415). For absolute MAP values, significant negative correlations occurred at pre, 0 min, 10 min, and 15 min (p ≤ 0.032 for all, ρ = −0.322 to −0.294), and a trend was seen at 5 min (p = 0.059, ρ = −0.272). There were no significant correlations for Δ HR (p = 1.000 for all, ρ = −0.026–0.123) or Δ MAP (p ≥ 0.270 for all, ρ = −0.199 to −0.040) (Supplementary Table S4).

### Interaction Analysis Between Sufentanil and Sevoflurane

**FIGURE 4:**
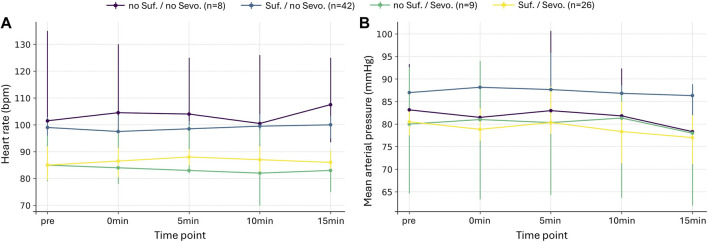
Interaction of sufentanil and sevoflurane Heart rate (bpm). **(A)** and mean arterial pressure (mmHg) **(B)** over time in four groups defined by the presence or absence of sufentanil (Suf.) and sevoflurane (Sevo.) administration. Values represent the group median at baseline (pre), during incision (0min), and 5, 10, and 15 min after incision. Vertical lines indicate 95% CI, displayed instead of IQR to allow precise interval estimates even in subgroups with small sample sizes.

Across all time points, no significant interaction effects between sufentanil and sevoflurane were observed for absolute values of HR (p ≥ 0.466 for all, partial η^2^ = 0.007–0.035) and MAP (p = 1.000 for all, partial η^2^ = 0.001–0.005) (Figures 4A,B; Supplementary Table S5). Analyses of relative changes yielded similar results, with no interaction between sufentanil and sevoflurane on the magnitude or direction of changes in Δ HR (p ≥ 0.887 for all, partial η^2^ = 0.000–0.018) or Δ MAP (p ≥ 0.453 for all, partial η^2^ = 0.003–0.031) (Supplementary Table S5).

### Effect of Norepinephrine on Hemodynamics

Since norepinephrine was the predominant catecholamine, and dosing of all catecholamines did not change significantly over time, subsequent analyses focused solely on the effects of norepinephrine. Partial correlation analyses revealed time-specific relationships between norepinephrine infusion rate and hemodynamics. After controlling for sufentanil and sevoflurane, norepinephrine positively correlated with HR in the early phase from pre to 5 min (p ≤ 0.038 for all, ρ = 0.287–0.318) and negatively with MAP during the later phase from 10 min to 15 min (p ≤ 0.032 for all, ρ = −0.303 to −0.294) (Supplementary Figures S1A, S1B; Supplementary Table S6). Thus, higher norepinephrine rates were associated with increased HR and decreased MAP at corresponding times. No significant partial correlations were found between sufentanil dose and HR or MAP after norepinephrine adjustment (p ≥ 0.547 for all, ρ = −0.048–0.175) (Supplementary Figures S1C, S1D; Supplementary Table S6), whereas the inverse relationship between sevoflurane concentration and HR remained significant throughout all time points (p < 0.001 for all, ρ = −0.502 to −0.414), and significant for MAP for 0 min, 10 min, and 15 min (p ≤ 0.033 for all, ρ = −0.316 to −0.292) (Supplementary Figures S1E, S1F; Supplementary Table S6).

## Discussion

This study is among the few to comprehensively analyze both the type and dosage of anesthetic agents and catecholamines during organ retrieval, along with their corresponding hemodynamic effects. Administration of anesthetic agents varied considerably among donors. Neither HR nor MAP showed a marked stress-related change in response to surgical stimulation, although MAP decreased slightly at 15 min compared with 5 minutes after incision. Sufentanil had no significant impact on hemodynamics. Sevoflurane was associated with consistently lower absolute HR and MAP values. However, compared with baseline, no significant differences were observed during or after incision. The hemodynamic effects of anesthetic agents remained unchanged after adjustment for concomitant use of norepinephrine.

### Hemodynamic Response During Organ Retrieval

Only few studies have systematically examined hemodynamic responses to surgical stimuli in DBD donors. The earliest available investigation reported an immediate increase in HR and MAP after incision in 10 donors but provided detailed information on anesthetic agent or catecholamine administration for only one case. In this donor, HR and MAP decreased 11 minutes after incision following administration of enflurane, while no opioids or catecholamines were given. In all other cases, only an unspecified reduction in catecholamine dosing was described, with HR and MAP normalizing within 25 min in all donors [13]. A subsequent case report documented similar increases in HR and MAP after incision in two donors but did not specify anesthetic or catecholamine use. Notably, only these two analyzed cases of a total of 30 reviewed DBD donors exhibited a hemodynamic response [11]. Another study involving 14 DBD donors reported elevations in HR and MAP 6 minutes after incision, without administration of anesthetic agents to any donor, but with dopamine in nine cases at constant doses [12]. Fitzgerald and colleagues reported on 11 DBD donors without anesthetic agents, seven of whom received dopamine. MAP increased immediately after incision and then decreased, while HR remained stable [9]. In a more recent study, donors with anesthetic agents exhibited higher HR and more frequent episodes of hypotension, although maximal MAP did not differ between groups. However, the study only distinguished between donors with and without anesthetic agent administration. Among those receiving agents (62% in total), the majority were given opioids alone (72%), while volatile anesthetics were administered in only 16% of cases. Additionally, hemodynamic parameters were not compared with baseline values prior to incision. Consequently, the observed hemodynamic effects cannot be attributed to any specific drug class or a possible reaction to surgical stimuli [4]. Taken together, these studies highlight the lack of standardized methodology for assessing hemodynamic parameters during organ retrieval, which are likely to contribute to inconsistent findings.

The observed hemodynamic responses in DBD donors have traditionally been interpreted as sympathetic reflexes to noxious stimuli mediated *via* spinal cord pathways in the absence of supraspinal control [20, 27]. Moreover, increases in systemic vascular resistance and endogen catecholamine levels after incision have been demonstrated [9, 12]. These findings have led to the hypothesis that anesthetic agents may attenuate such hemodynamic responses and thereby exert organ-protective effects [6].

Our findings add new evidence to this conflicting body of literature. The total cohort in our study exhibited no increase in HR or MAP over time or after incision compared with baseline. Sufentanil did not alter hemodynamic values. There was even a non-significant trend to higher MAP values in donors receiving sufentanil, which aligns with results from previous studies [18, 19]. Thus, our results do not support the use of opioids for hemodynamic control in DBD donors, contrary to prior recommendations [20].

Opioid receptors are widely distributed within the central nervous system (CNS), including the substantia gelatinosa of the dorsal horn and to a lesser extent in peripheral tissues. Within the CNS, activation of opioid receptors in the midbrain is thought to be a major mechanism of opioid-induced analgesia. They stimulate descending inhibitory pathways, which results in a reduction of nociceptive transmission from the periphery to the thalamus [28]. Peripheral opioid effects are variable and are primarily observed under conditions of tissue injury, such as inflammation or neuropathy [29]. Although opioids can exert direct inhibitory effects when administered epidural, spinal or peripheral, clinically relevant analgesia following systemic administration seems to be predominantly mediated *via* central mechanisms [28]. In DBD donors, the absence of supraspinal integration and functioning descending inhibitory pathways likely limits the ability of systemically administered opioids to attenuate nociceptive transmission and autonomic responses. Moreover, the absence of effective cerebral perfusion in the brain-dead state prevents systemically administered opioids from reaching the brain, rendering potential central antinociceptive and reflex-modulating effects unlikely [6]. Hence, the isolated use of opioids during organ retrieval appears ineffective for controlling possible neurovegetative reflexes or conferring tissue protection [4]. In contrast, sevoflurane significantly decreased HR and MAP, but these characteristics were already seen at baseline and were independent of surgical stimuli. This pattern is consistent with the general pharmacodynamic properties of volatile anesthetics, which induce vasodilation predominantly through actions at the spinal cord level, independent of any effects on the CNS [30], and may also involve direct myocardial effects or reductions in systemic vascular resistance [31]. Importantly, this effect does not reflect modulation of responses to surgical stimuli.

### Organ Protective Effects of Anesthetic Agents

Administering anesthetic agents during organ retrieval could nonetheless be considered an anesthetic preconditioning strategy to attenuate IRI. Several studies have suggested that volatile anesthetics exert cardioprotective effects in cardiac surgery [32]. Under brain death conditions, these mechanisms have only been investigated scarcely. Experimental data suggest that pharmacological modulation could possibly mitigate IRI [33], and volatile anesthetics have been proposed to exert similar protective effects through distinct pathways [14, 17]. One single clinical study showed that preconditioning with sevoflurane during organ retrieval improved liver graft function [34], whereas a later study was unable to confirm a protective effect in early or long-term graft survival for kidney, liver, lung, or heart transplantation [16]. Another study likewise found no association between the use of volatile anesthetics and/or opioids and kidney graft function [4]. Overall, both approaches - administering or omitting anesthetic agents - lack robust clinical evaluation and high-quality evidence regarding their impact on graft outcomes [4].

### Hemodynamic Management During Organ Retrieval

Norepinephrine was the predominant catecholamine used in our cohort, while other catecholamines were administered in only a few donors, consistent with previous findings [4, 8]. Whether their use was intended as part of an organ-protective strategy, for hemodynamic stabilization, or simply continued from preexisting ICU therapy cannot be determined in the retrospective setting of our study. Overall, catecholamine dosing did not change significantly over time, and a MAP ≥65 mmHg was maintained in most donors throughout the observation period.

Very few other studies have investigated hemodynamic management during organ retrieval. One study reported that the target MAP of 65 mmHg was not maintained for a considerable period in 62% of DBD donors [4]. In another study, 24% of donors experienced hypotension lasting from a minimum of 10 min to a maximum of 96 min [8]. Interestingly, our data show that shortly after incision, a decline in MAP was particularly evident in the cohort receiving VA. Notably, higher norepinephrine doses correlated with lower MAP values, likely reflecting the reactive use of vasopressors in response to hypotension rather than a direct hypotensive effect of norepinephrine.

This phenomenon is consistent with earlier studies demonstrating a rapid decline in endogenous catecholamine levels shortly after incision [9, 11]. The combination of reduced catecholamine concentrations and the vasodilatory properties of volatile anesthetics may increase the risk of hypotension and should therefore be carefully considered when volatile anesthetics are administered. Opioids, in contrast, did not contribute to hemodynamic modulation in our cohort.

Continuous waveform monitoring enables real-time detection of hemodynamic changes in critically ill patients and is essential for timely intervention [35]. Its use in DBD donors has not been described in the literature but may represent a promising approach to ensure hemodynamic stability during organ retrieval and to improve understanding of the effects of anesthetic agents and catecholamines in this setting.

### Ethical Considerations

Beyond physiological considerations, the choice to administer anesthetic agents may reflect clinicians’ unease with the concept of brain death—a difficulty frequently noted in the literature [24, 25]. Administering anesthesia to a DBD donor for reasons other than a potential protective effect risks undermining the conceptual and ethical clarity of brain death and could have detrimental implications for both public perception and healthcare professionals [36]. Consequently, Turner suggested that the term ‘anesthesia´ itself should perhaps be avoided in this context to minimize misunderstanding of the transplantation process [37].

### Limitations

Potential confounders could not be controlled for, such as details of preceding ICU management strategies or preexisting cardiovascular or other organ specific conditions. Hemodynamic measurements were referenced to the time of the first incision, which may not correspond to the most relevant noxious stimulus. The administration of other drugs (e.g., corticosteroids) or fluids was not considered, although these may have influenced hemodynamic responses to an unknown extent. As a single-center study, generalizability is limited, and management strategies may also have evolved over the long observation period, introducing potential heterogeneity. The decision to administer anesthetic agents was not randomized and may have been influenced by clinical judgement or donor characteristics. Finally, graft outcomes could not be analyzed. Therefore, no conclusions can be drawn regarding the potential effect of anesthetic management on transplantation results.

## Conclusion

Anesthetic management of organ retrieval varied considerably. Neither the surgical stimulus nor sufentanil had a measurable impact on hemodynamics in DBD donors. This suggests that opioids are not indicated during organ retrieval until further evidence about other pharmacological organ-protective effects is available. Sevoflurane was associated with consistently lower absolute HR and MAP values, but this was independent of the surgical stimulus. These effects of volatile anesthetics in DBD donors must be carefully considered to maintain hemodynamic stability. Thus, our findings highlight two key points. First, there is an urgent need for evidence-based recommendations on anesthetic management during organ retrieval, encompassing both pharmacological and ethical considerations. Second, such recommendations should be based on future research elucidating the relationship between anesthetic management and graft outcomes. This may represent a promising strategy to enhance organ-protective measures and should be evaluated in prospective studies to determine whether it ultimately improves transplant outcomes.

## Data Availability

The data analyzed in this study is subject to the following licenses/restrictions: Anonymized raw data supporting the conclusions of this article will be made available on reasonable request. Requests to access these datasets should be directed to jan.englbrecht@ukmuenster.de.

## Supplementary Material

SUPPLEMENTARY FIGURE S1 Partial correlation analyses between norepinephrine, sufentanil, sevoflurane and hemodynamic parameters. Scatterplots display the relationship between norepinephrine (µg kg^−1^ min^−1^) and heart rate (HR, bpm) **(A)** and mean arterial pressure (MAP, mmHg) **(B)**; sufentanil (µg kg^−1^) and HR **(C)** and MAP **(D)**; sevoflurane (et %) and HR **(E)** and MAP **(F)**. Data are shown for baseline (pre), during incision (0 min), and 5, 10, and 15 minutes after incision. For each time point, individual data points represent partial residuals of HR and MAP after statistical adjustment for both sufentanil and sevoflurane **(A,B)**, and norepinephrine **(C–F)**, respectively. Each panel contains a linear regression line, and a shaded band depicts the 95% CI. Raw predictor values were plotted against partial residuals of HR or MAP obtained after adjustment for covariates. Accordingly, x-axes display variables in their original physical units, while y-axes represent dimensionless residuals reflecting adjusted deviations from model-predicted values.
