# Supplementary material for: Anesthetic Management of Brain-Dead Donors During Organ Retrieval: Hemodynamic Effects and Potential Organ-Protective Implications – A Retrospective Analysis of 85 Cases
Source: Transpl Int. 2026 Apr 21;39:16262. doi: 10.3389/ti.2026.16262 (PMC13139039; doi:10.3389/ti.2026.16262)
Supplement: Supplementary file 1 [file Table1.docx]

**Supplementary Material**

Effect sizes were calculated depending on test type. Kendall’s W for Friedman tests, Wilcoxon r for paired non-parametric comparisons, rank-biserial r for Mann–Whitney U tests, Spearman’s ρ (or partial Spearman’s ρ when adjusted for confounders) for correlation analyses, Cohen’s d_z_ for paired t-tests, and partial η² for ANOVA-based models.

**Supplemental Table 1. Infusion rates of catecholamines over time**

| Drug | Test | Statistic | p |  | Effect size |
| --- | --- | --- | --- | --- | --- |
| Norepinephrine | Friedman | χ² (4)=7.54 | 0.110 |  | Kendall´s W=0.028 |
| Epinephrine | Friedman | χ² (4)=1.71 | 0.788 |  | Kendall´s W=0.086 |
| Dopamine | Friedman | χ² (4)=0.00 | 1.000 |  | Kendall´s W=0.000 |
| Dobutamine | Friedman | χ² (4)=0.00 | 1.000 |  | Kendall´s W=0.000 |
| Vasopressin | RM-ANOVA | F (4,64)=3.31 | **0.016** |  | partial η²=0.171 |
| Post hoc Vasopressin | | | | |  |
| Comparison | **Test** | **Statistic** | **p** | **p (Bonf.)** |  |
| Vasopressin 0min vs pre | paired t-test | t (16)=1.00 | 0.332 | 1.000 | Cohen´s d_z_=0.243 |
| Vasopressin 5min vs pre | paired t-test | t (16)=1.81 | 0.090 | 0.359 | Cohen´s d_z_=0.439 |
| Vasopressin 10min vs pre | paired t-test | t (16)=2.11 | 0.051 | 0.206 | Cohen´s d_z_=0.512 |
| Vasopressin 15min vs pre | paired t-test | t (16)=2.11 | 0.051 | 0.206 | Cohen´s d_z_=0.512 |

RM-ANOVA: repeated measure ANOVA, Bonf.: Bonferroni corrected

**Supplemental Table 2. Hemodynamics over time**

| Param. | Comparison | Test | Statistic | p | p (Bonf.) | Effect size |
| --- | --- | --- | --- | --- | --- | --- |
| HR | Across time (pre, 0, 5, 10, 15min) | Friedman | χ² (4)=4.807 | 0.308 | 0.308 | Kendall´s W=0.014 |
| HR | pre vs 0min | Wilcoxon signed-rank | W=1088 | 0.910 | 1.000 | r=0.012 |
| HR | pre vs 5min | Wilcoxon signed-rank | W=924 | 0.246 | 1.000 | r=0.126 |
| HR | pre vs 10min | Wilcoxon signed-rank | W=902 | 0.192 | 1.000 | r=0.142 |
| HR | pre vs 15min | Wilcoxon signed-rank | W=1133 | 0.086 | 0.862 | r=0.186 |
| HR | 0min vs 5min | Wilcoxon signed-rank | W=980 | 0.416 | 1.000 | r=0.088 |
| HR | 0min vs 10min | Wilcoxon signed-rank | W=874 | 0.191 | 1.000 | r=0.142 |
| HR | 0min vs 15min | Wilcoxon signed-rank | W=920 | 0.085 | 0.850 | r=0.187 |
| HR | 5min vs 10min | Wilcoxon signed-rank | W=830 | 0.302 | 1.000 | r=0.122 |
| HR | 5min vs 15min | Wilcoxon signed-rank | W=1050 | 0.577 | 1.000 | r=0.061 |
| HR | 10min vs 15min | Wilcoxon signed-rank | W=670 | 0.213 | 1.000 | r=0.135 |
| MAP | Across time (pre, 0, 5, 10, 15min) | Friedman | χ² (4)=14.143 | 0.007 | **0.007** | Kendall´s W=0.042 |
| MAP | pre vs 0min | Wilcoxon signed-rank | W=1560 | 0.775 | 1.000 | r=0.031 |
| MAP | pre vs 5min | Wilcoxon signed-rank | W=1298 | 0.062 | 0.621 | r=0.202 |
| MAP | pre vs 10min | Wilcoxon signed-rank | W=1569 | 0.429 | 1.000 | r=-0.086 |
| MAP | pre vs 15min | Wilcoxon signed-rank | W=1419 | 0.103 | 1.000 | r=-0.177 |
| MAP | 0min vs 5min | Wilcoxon signed-rank | W=1072 | 0.043 | 0.432 | r=0.219 |
| MAP | 0min vs 10min | Wilcoxon signed-rank | W=1498 | 0.560 | 1.000 | r=0.063 |
| MAP | 0min vs 15min | Wilcoxon signed-rank | W=1252 | 0.109 | 1.000 | r=-0.174 |
| MAP | 5min vs 10min | Wilcoxon signed-rank | W=1114 | 0.023 | 0.229 | r=-0.247 |
| MAP | 5min vs 15min | Wilcoxon signed-rank | W=1010 | 0.003 | **0.034** | r=-0.317 |
| MAP | 10min vs 15min | Wilcoxon signed-rank | W=1082 | 0.049 | 0.488 | r=-0.214 |

Param.: Parameter, HR: heartrate, MAP: mean arterial pressure, Bonf.: Bonferroni corrected

**Supplemental Table 3. Effects of sufentanil on heart rate and mean arterial pressure**

| Analysis | Param. | Comparison_Predictor | Test | Statistic | p | p (Bonf.) | Effect size |
| --- | --- | --- | --- | --- | --- | --- | --- |
| Sufentanil (b/g) | HR | With vs Without (absolute) – pre | Mann–Whitney | U=592 | 0.882 | 1.000 | r=0.024 |
| Sufentanil (b/g) | HR | With vs Without (absolute) – 0min | Mann–Whitney | U=588 | 0.912 | 1.000 | r=0.018 |
| Sufentanil (b/g) | HR | With vs Without (absolute) – 5min | Mann–Whitney | U=584 | 0.947 | 1.000 | r=0.011 |
| Sufentanil (b/g) | HR | With vs Without (absolute) – 10min | Mann–Whitney | U=560 | 0.852 | 1.000 | r=-0.030 |
| Sufentanil (b/g) | HR | With vs Without (absolute) – 15min | Mann–Whitney | U=525 | 0.564 | 1.000 | r=-0.092 |
| Sufentanil (b/g) | HR | With vs Without (Δ vs pre) – 0min | Mann–Whitney | U=536 | 0.645 | 1.000 | r=-0.073 |
| Sufentanil (b/g) | HR | With vs Without (Δ vs pre) – 5min | Mann–Whitney | U=615 | 0.686 | 1.000 | r=0.064 |
| Sufentanil (b/g) | HR | With vs Without (Δ vs pre) – 10min | Mann–Whitney | U=576 | 0.987 | 1.000 | r=-0.003 |
| Sufentanil (b/g) | HR | With vs Without (Δ vs pre) – 15min | Mann–Whitney | U=511 | 0.463 | 1.000 | r=-0.116 |
| Sufentanil (b/g) | MAP | With vs Without (absolute) – pre | Mann–Whitney | U=488 | 0.328 | 1.000 | r=-0.155 |
| Sufentanil (b/g) | MAP | With vs Without (absolute) – 0min | Mann–Whitney | U=502 | 0.407 | 1.000 | r=-0.131 |
| Sufentanil (b/g) | MAP | With vs Without (absolute) – 5min | Mann–Whitney | U=448 | 0.155 | 0.774 | r=-0.225 |
| Sufentanil (b/g) | MAP | With vs Without (absolute) – 10min | Mann–Whitney | U=452 | 0.168 | 0.839 | r=-0.218 |
| Sufentanil (b/g) | MAP | With vs Without (absolute) – 15min | Mann–Whitney | U=412 | 0.069 | 0.345 | r=-0.287 |
| Sufentanil (b/g) | MAP | With vs Without (Δ vs pre) – 0min | Mann–Whitney | U=469 | 0.233 | 0.932 | r=-0.189 |
| Sufentanil (b/g) | MAP | With vs Without (Δ vs pre) – 5min | Mann–Whitney | U=476 | 0.265 | 1.000 | r=-0.176 |
| Sufentanil (b/g) | MAP | With vs Without (Δ vs pre) – 10min | Mann–Whitney | U=446 | 0.150 | 0.600 | r=-0.228 |
| Sufentanil (b/g) | MAP | With vs Without (Δ vs pre) – 15min | Mann–Whitney | U=488 | 0.325 | 1.000 | r=-0.156 |
| Sufentanil (corr.) | HR | dose vs absolute – pre | Spearman | ρ=0.047 | 0.672 | 1.000 |  |
| Sufentanil (corr.) | HR | dose vs absolute – 0min | Spearman | ρ=0.058 | 0.599 | 1.000 |  |
| Sufentanil (corr.) | HR | dose vs absolute – 5min | Spearman | ρ=0.050 | 0.649 | 1.000 |  |
| Sufentanil (corr.) | HR | dose vs absolute – 10min | Spearman | ρ=0.077 | 0.486 | 1.000 |  |
| Sufentanil (corr.) | HR | dose vs absolute – 15min | Spearman | ρ=0.157 | 0.151 | 0.754 |  |
| Sufentanil (corr.) | HR | dose vs Δ – 0min | Spearman | ρ=0.083 | 0.453 | 1.000 |  |
| Sufentanil (corr.) | HR | dose vs Δ – 5min | Spearman | ρ=-0.073 | 0.508 | 1.000 |  |
| Sufentanil (corr.) | HR | dose vs Δ – 10min | Spearman | ρ=0.017 | 0.877 | 1.000 |  |
| Sufentanil (corr.) | HR | dose vs Δ – 15min | Spearman | ρ=0.132 | 0.230 | 0.919 |  |
| Sufentanil (corr.) | MAP | dose vs absolute – pre | Spearman | ρ=-0.073 | 0.507 | 1.000 |  |
| Sufentanil (corr.) | MAP | dose vs absolute – 0min | Spearman | ρ=-0.003 | 0.975 | 1.000 |  |
| Sufentanil (corr.) | MAP | dose vs absolute – 5min | Spearman | ρ=0.029 | 0.796 | 1.000 |  |
| Sufentanil (corr.) | MAP | dose vs absolute – 10min | Spearman | ρ=0.089 | 0.418 | 1.000 |  |
| Sufentanil (corr.) | MAP | dose vs absolute – 15min | Spearman | ρ=0.138 | 0.207 | 1.000 |  |
| Sufentanil (corr.) | MAP | dose vs Δ – 0min | Spearman | ρ=0.252 | 0.020 | 0.079 |  |
| Sufentanil (corr.) | MAP | dose vs Δ – 5min | Spearman | ρ=0.125 | 0.254 | 1.000 |  |
| Sufentanil (corr.) | MAP | dose vs Δ – 10min | Spearman | ρ=0.245 | 0.024 | 0.094 |  |
| Sufentanil (corr.) | MAP | dose vs Δ – 15min | Spearman | ρ=0.233 | 0.032 | 0.128 |  |

Param.: parameter, b/g: between groups, corr.: correlation, HR: heart rate, MAP: mean arterial pressure, Bonf.: Bonferroni corrected

**Supplemental Table 4. Effects of volatiles on heart rate and mean arterial pressure**

| Analysis | Param. | Comparison_Predictor | Test | Statistic | p | p (Bonf.) | Effect size |
| --- | --- | --- | --- | --- | --- | --- | --- |
| Sevo. (b/g) | HR | With vs Without (absolute) – pre | Mann–Whitney | U=1290 | 0.000 | **0.001** | r=0.474 |
| Sevo. (b/g) | HR | With vs Without (absolute) – 0min | Mann–Whitney | U=1286 | 0.000 | **0.001** | r=0.470 |
| Sevo. (b/g) | HR | With vs Without (absolute) – 5min | Mann–Whitney | U=1293 | 0.000 | **0.001** | r=0.478 |
| Sevo. (b/g) | HR | With vs Without (absolute) – 10min | Mann–Whitney | U=1318 | 0.000 | **0.000** | r=0.506 |
| Sevo. (b/g) | HR | With vs Without (absolute) – 15min | Mann–Whitney | U=1374 | 0.000 | **0.000** | r=0.571 |
| Sevo. (b/g) | HR | With vs Without (Δ vs pre) – 0min | Mann–Whitney | U=904 | 0.801 | 1.000 | r=0.033 |
| Sevo. (b/g) | HR | With vs Without (Δ vs pre) – 5min | Mann–Whitney | U=775 | 0.371 | 1.000 | r=-0.114 |
| Sevo. (b/g) | HR | With vs Without (Δ vs pre) – 10min | Mann–Whitney | U=913 | 0.736 | 1.000 | r=0.043 |
| Sevo. (b/g) | HR | With vs Without (Δ vs pre) – 15min | Mann–Whitney | U=917 | 0.710 | 1.000 | r=0.048 |
| Sevo. (b/g) | MAP | With vs Without (absolute) – pre | Mann–Whitney | U=1139 | 0.019 | 0.093 | r=0.302 |
| Sevo. (b/g) | MAP | With vs Without (absolute) – 0min | Mann–Whitney | U=1197 | 0.004 | **0.020** | r=0.386 |
| Sevo. (b/g) | MAP | With vs Without (absolute) – 5min | Mann–Whitney | U=1130 | 0.023 | 0.114 | r=0.292 |
| Sevo. (b/g) | MAP | With vs Without (absolute) – 10min | Mann–Whitney | U=1148 | 0.015 | **0.075** | r=0.312 |
| Sevo. (b/g) | MAP | With vs Without (absolute) – 15min | Mann–Whitney | U=1159 | 0.011 | **0.057** | r=0.325 |
| Sevo. (b/g) | MAP | With vs Without (Δ vs pre) – 0min | Mann–Whitney | U=1096 | 0.048 | 0.193 | r=0.253 |
| Sevo. (b/g) | MAP | With vs Without (Δ vs pre) – 5min | Mann–Whitney | U=902 | 0.816 | 1.000 | r=0.030 |
| Sevo. (b/g) | MAP | With vs Without (Δ vs pre) – 10min | Mann–Whitney | U=978 | 0.362 | 1.000 | r=0.117 |
| Sevo. (b/g) | MAP | With vs Without (Δ vs pre) – 15min | Mann–Whitney | U=948 | 0.517 | 1.000 | r=0.083 |
| Sevo. (corr.) | HR | conc. vs absolute – pre | Spearman | ρ=-0.425 | 0.000 | **0.000** |  |
| Sevo. (corr.) | HR | conc. vs absolute – 0min | Spearman | ρ=-0.415 | 0.000 | **0.000** |  |
| Sevo. (corr.) | HR | conc. vs absolute – 5min | Spearman | ρ=-0.425 | 0.000 | **0.000** |  |
| Sevo. (corr.) | HR | conc. vs absolute – 10min | Spearman | ρ=-0.448 | 0.000 | **0.000** |  |
| Sevo. (corr.) | HR | conc. vs absolute – 15min | Spearman | ρ=-0.499 | 0.000 | **0.000** |  |
| Sevo. (corr.) | HR | conc. vs Δ – 0min | Spearman | ρ=0.002 | 0.987 | 1.000 |  |
| Sevo. (corr.) | HR | conc. vs Δ – 5min | Spearman | ρ=0.123 | 0.263 | 1.000 |  |
| Sevo. (corr.) | HR | conc. vs Δ – 10min | Spearman | ρ=-0.003 | 0.981 | 1.000 |  |
| Sevo. (corr.) | HR | conc. vs Δ – 15min | Spearman | ρ=-0.026 | 0.813 | 1.000 |  |
| Sevo. (corr.) | MAP | conc. vs absolute – pre | Spearman | ρ=-0.294 | 0.006 | **0.032** |  |
| Sevo. (corr.) | MAP | conc. vs absolute – 0min | Spearman | ρ=-0.332 | 0.002 | **0.010** |  |
| Sevo. (corr.) | MAP | conc. vs absolute – 5min | Spearman | ρ=-0.272 | 0.012 | 0.059 |  |
| Sevo. (corr.) | MAP | conc. vs absolute – 10min | Spearman | ρ=-0.318 | 0.003 | **0.015** |  |
| Sevo. (corr.) | MAP | conc. vs absolute – 15min | Spearman | ρ=-0.322 | 0.003 | **0.013** |  |
| Sevo. (corr.) | MAP | conc. vs Δ – 0min | Spearman | ρ=-0.199 | 0.067 | 0.270 |  |
| Sevo. (corr.) | MAP | conc. vs Δ – 5min | Spearman | ρ=-0.040 | 0.714 | 1.000 |  |
| Sevo. (corr.) | MAP | conc. vs Δ – 10min | Spearman | ρ=-0.145 | 0.186 | 0.745 |  |
| Sevo. (corr.) | MAP | conc. vs Δ – 15min | Spearman | ρ=-0.086 | 0.434 | 1.000 |  |

Sevo.: sevoflurane, b/g: between groups, corr.: correlation, HR: heart rate, MAP: mean arterial pressure, Bonf.: Bonferroni corrected

**Supplemental Table 5. Interaction analysis between sufentanil and sevoflurane**

| Param. | Analysis | Time | Predictor | Test | Statistic | p | p (Bonf.) | Effect size |
| --- | --- | --- | --- | --- | --- | --- | --- | --- |
| HR | Absolute | pre | Suf*Sevo | Two-way ANOVA | F (1, 81) =0.763 | 0.385 | 1.000 | partial η²=0.009 |
| HR | Absolute | 0min | Suf*Sevo | Two-way ANOVA | F (1, 81) =1.156 | 0.285 | 1.000 | partial η²=0.014 |
| HR | Absolute | 5min | Suf*Sevo | Two-way ANOVA | F (1, 81) =1.003 | 0.320 | 1.000 | partial η²=0.012 |
| HR | Absolute | 10min | Suf*Sevo | Two-way ANOVA | F (1, 81) =0.582 | 0.448 | 1.000 | partial η²=0.007 |
| HR | Absolute | 15min | Suf*Sevo | Two-way ANOVA | F (1, 81) =2.959 | 0.089 | 0.446 | partial η²=0.035 |
| HR | Δ vs pre | 0min | Suf*Sevo | Two-way ANOVA | F (1, 81) =0.222 | 0.639 | 1.000 | partial η²=0.003 |
| HR | Δ vs pre | 5min | Suf*Sevo | Two-way ANOVA | F (1, 81) =0.000 | 0.992 | 1.000 | partial η²=0.000 |
| HR | Δ vs pre | 10min | Suf*Sevo | Two-way ANOVA | F (1, 81) =0.159 | 0.691 | 1.000 | partial η²=0.002 |
| HR | Δ vs pre | 15min | Suf*Sevo | Two-way ANOVA | F (1, 81) =1.517 | 0.222 | 0.887 | partial η²=0.018 |
| MAP | Absolute | Pre | Suf*Sevo | Two-way ANOVA | F (1, 81) =0.062 | 0.805 | 1.000 | partial η²=0.001 |
| MAP | Absolute | 0min | Suf*Sevo | Two-way ANOVA | F (1, 81) =0.402 | 0.528 | 1.000 | partial η²=0.005 |
| MAP | Absolute | 5min | Suf*Sevo | Two-way ANOVA | F (1, 81) =0.005 | 0.944 | 1.000 | partial η²=0.000 |
| MAP | Absolute | 10min | Suf*Sevo | Two-way ANOVA | F (1, 81) =0.202 | 0.654 | 1.000 | partial η²=0.002 |
| MAP | Absolute | 15min | Suf*Sevo | Two-way ANOVA | F (1, 81) =0.077 | 0.782 | 1.000 | partial η²=0.001 |
| MAP | Δ vs pre | 0min | Suf*Sevo | Two-way ANOVA | F (1, 81) =2.563 | 0.113 | 0.453 | partial η²=0.031 |
| MAP | Δ vs pre | 5min | Suf*Sevo | Two-way ANOVA | F (1, 81) =0.245 | 0.622 | 1.000 | partial η²=0.003 |
| MAP | Δ vs pre | 10min | Suf*Sevo | Two-way ANOVA | F (1, 81) =0.626 | 0.431 | 1.000 | partial η²=0.008 |
| MAP | Δ vs pre | 15min | Suf*Sevo | Two-way ANOVA | F (1, 81) =0.270 | 0.604 | 1.000 | partial η²=0.003 |

Param.: parameter, HR: heart rate, MAP: mean arterial pressure, Suf: sufentanil, Sevo: sevoflurane, Bonf.: Bonferroni corrected

**Supplemental Table 6. Partial correlation analyses of relationships between norepinephrine and hemodynamics after controlling for sufentanil and sevoflurane, and vice versa**

| Analysis (partial) | Param. | Comparison_Predictor | Test | Statistic | p | p (Bonf.) |
| --- | --- | --- | --- | --- | --- | --- |
| Norepinephrine | HR | NE → HR (time pre) \| control: Suf/Sevo | Spearman partial | ρ=0.318 | 0.003 | **0.015** |
| Norepinephrine | HR | NE → HR (time 0min) \| control: Suf/Sevo | Spearman partial | ρ=0.309 | 0.004 | **0.020** |
| Norepinephrine | HR | NE → HR (time 5min) \| control: Suf/Sevo | Spearman partial | ρ=0.287 | 0.008 | **0.038** |
| Norepinephrine | HR | NE → HR (time 10min) \| control: Suf/Sevo | Spearman partial | ρ=0.218 | 0.045 | 0.223 |
| Norepinephrine | HR | NE → HR (time 15min) \| control: Suf/Sevo | Spearman partial | ρ=0.235 | 0.030 | 0.152 |
| Norepinephrine | MAP | NE → MAP (time pre) \| control: Suf/Sevo | Spearman partial | ρ=-0.112 | 0.306 | 1.000 |
| Norepinephrine | MAP | NE → MAP (time 0min) \| control: Suf/Sevo | Spearman partial | ρ=-0.186 | 0.088 | 0.438 |
| Norepinephrine | MAP | NE → MAP (time 5min) \| control: Suf/Sevo | Spearman partial | ρ=-0.259 | 0.017 | 0.084 |
| Norepinephrine | MAP | NE → MAP (time 10min) \| control: Suf/Sevo | Spearman partial | ρ=-0.303 | 0.005 | **0.024** |
| Norepinephrine | MAP | NE → MAP (time 15min) \| control: Suf/Sevo | Spearman partial | ρ=-0.294 | 0.006 | **0.032** |
| Sevoflurane | HR | Sevo conc. → HR (time pre) \| control: NE | Spearman partial | ρ=-0.427 | 0.000 | **0.000** |
| Sevoflurane | HR | Sevo conc. → HR (time 0min) \| control: NE | Spearman partial | ρ=-0.419 | 0.000 | **0.000** |
| Sevoflurane | HR | Sevo conc. → HR (time 5min) \| control: NE | Spearman partial | ρ=-0.414 | 0.000 | **0.000** |
| Sevoflurane | HR | Sevo conc. → HR (time 10min) \| control: NE | Spearman partial | ρ=-0.438 | 0.000 | **0.000** |
| Sevoflurane | HR | Sevo conc. → HR (time 15min) \| control: NE | Spearman partial | ρ=-0.502 | 0.000 | **0.000** |
| Sevoflurane | MAP | Sevo conc. → MAP (time pre) \| control: NE | Spearman partial | ρ=-0.276 | 0.010 | 0.052 |
| Sevoflurane | MAP | Sevo conc. → MAP (time 0min) \| control: NE | Spearman partial | ρ=-0.316 | 0.003 | **0.016** |
| Sevoflurane | MAP | Sevo conc. → MAP (time 5min) \| control: NE | Spearman partial | ρ=-0.259 | 0.016 | 0.082 |
| Sevoflurane | MAP | Sevo conc. → MAP (time 10min) \|control: NE | Spearman partial | ρ=-0.315 | 0.003 | **0.017** |
| Sevoflurane | MAP | Sevo conc. → MAP (time 15min) \|control: NE | Spearman partial | ρ=-0.292 | 0.007 | **0.033** |
| Sufentanil | HR | Suf dose → HR (time pre) \| control: NE | Spearman partial | ρ=0.062 | 0.572 | 1.000 |
| Sufentanil | HR | Suf dose → HR (time 0min) \| control: NE | Spearman partial | ρ=0.050 | 0.649 | 1.000 |
| Sufentanil | HR | Suf dose → HR (time 5min) \| control: NE | Spearman partial | ρ=0.080 | 0.467 | 1.000 |
| Sufentanil | HR | Suf dose → HR (time 10min) \| control: NE | Spearman partial | ρ=0.085 | 0.437 | 1.000 |
| Sufentanil | HR | Suf dose → HR (time 15min) \| control: NE | Spearman partial | ρ=0.175 | 0.109 | 0.547 |
| Sufentanil | MAP | Suf dose → MAP (time pre) \| control: NE | Spearman partial | ρ=-0.048 | 0.661 | 1.000 |
| Sufentanil | MAP | Suf dose → MAP (time 0min) \| control: NE | Spearman partial | ρ=-0.022 | 0.839 | 1.000 |
| Sufentanil | MAP | Suf dose → MAP (time 5min) \| control: NE | Spearman partial | ρ=0.049 | 0.653 | 1.000 |
| Sufentanil | MAP | Suf dose → MAP (time 10min) \| control: NE | Spearman partial | ρ=0.106 | 0.333 | 1.000 |
| Sufentanil | MAP | Suf dose → MAP (time 15min) \| control: NE | Spearman partial | ρ=0.162 | 0.139 | 0.697 |

Param.: parameter, HR: heart rate, MAP: mean arterial pressure, NE: norepinephrine, Suf: sufentanil, Sevo: sevoflurane, Bonf.: Bonferroni corrected
