# Supplementary figures and images for: Anesthetic Management of Brain-Dead Donors During Organ Retrieval: Hemodynamic Effects and Potential Organ-Protective Implications – A Retrospective Analysis of 85 Cases
Source: Transpl Int. 2026 Apr 21;39:16262. doi: 10.3389/ti.2026.16262 (PMC13139039; doi:10.3389/ti.2026.16262)

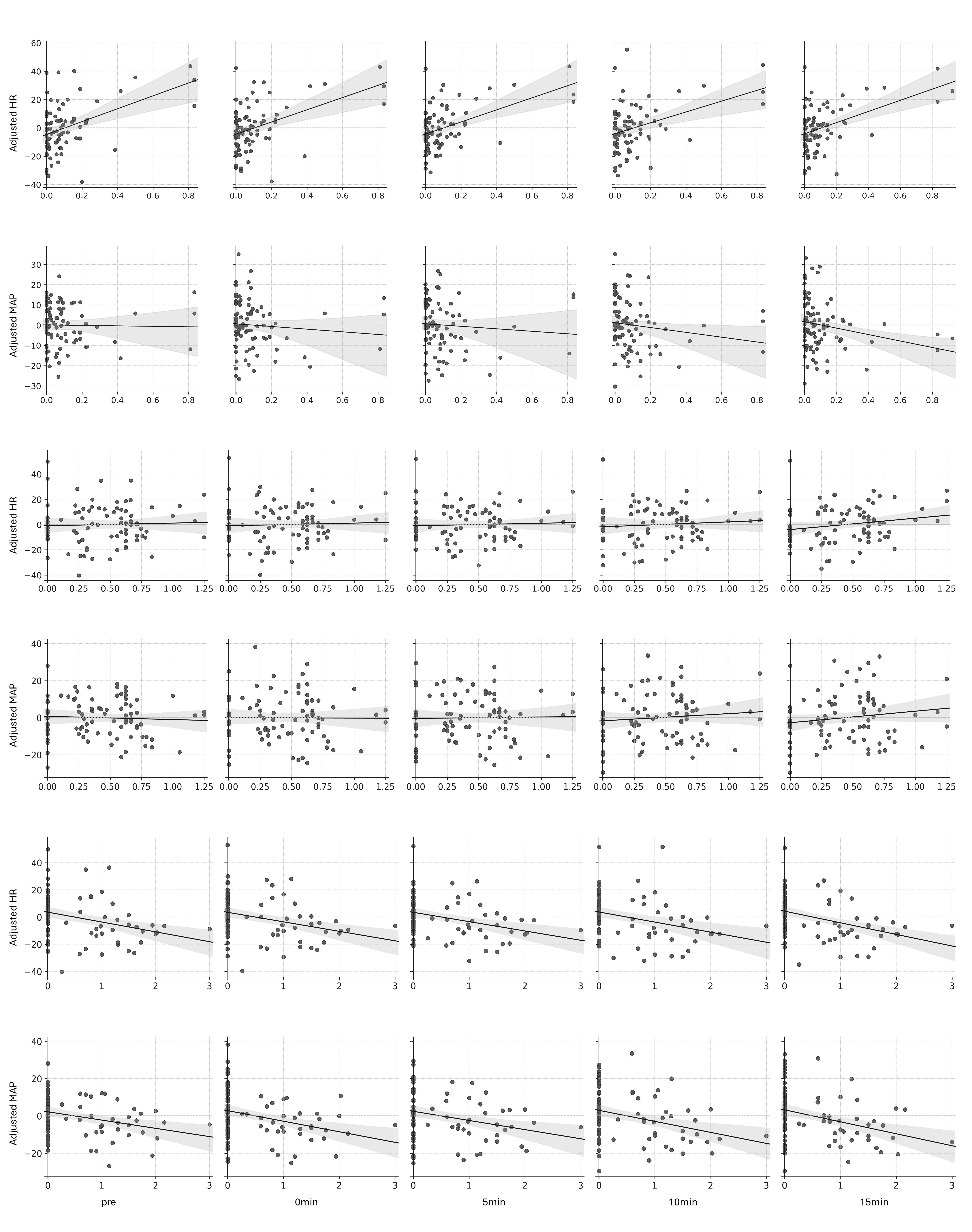

Supplement: Supplementary file 2 [file Image1.JPEG]
